# IL-17A-producing γδ T cells and classical monocytes are associated with a rapid alloimmune response following vascularized composite allotransplantation in mice

**DOI:** 10.3389/fimmu.2025.1584916

**Published:** 2025-06-04

**Authors:** Tetsuya Tajima, Wenming Zhang, Shuling Han, Andrea Reitsma, James T. Harden, Samuel Fuentes, Ayaka Sonehara, Carlos O. Esquivel, Olivia M. Martinez, Sheri M. Krams

**Affiliations:** ^1^ Department of Surgery, Division of Abdominal Transplantation, Stanford University School of Medicine, Stanford, CA, United States; ^2^ Stanford Immunology, Stanford University School of Medicine, Stanford, CA, United States

**Keywords:** vascularized composite allotransplantation, γδ T cell, classical monocyte, IL-17, mouse, IFN-γ, perforin, granzyme

## Abstract

**Background:**

Vascularized Composite Allotransplantation (VCA) is an important therapeutic option for patients that incur debilitating injuries to the face or limbs. The complexity and immunogenicity of tissue types within VCA grafts pose unique challenges and necessitate the use of intensive immunosuppression; however, graft rejection remains a challenge in VCA.

**Methods:**

Deep proteomic profiling and high dimensional analysis with cytometry time of flight were used to define the cell types and effector mechanisms elicited by VCA in BALB/c (H-2Kd) > C57BL/6 (H-2Kb) limb recipients. Spleen and cervical draining lymph nodes were collected post-transplant days 1, 3, 5, and 7 (n =4–6 mice/group/day). We identified dynamic, coordinated signatures in T cell and monocyte populations associated with VCA allograft rejection.

**Results:**

In comparison to syngeneic transplant recipients, allogeneic recipients exhibited significant alterations in the immune cell populations within secondary lymphoid tissues. These changes included very early expansion of double-negative TCRβ^-^ T cells, including IL-17A-producing γδ T cells, and patrolling monocytes. Subsequently, CD8+CD62L+ T cells and CD8+ effector/effector memory T cells (Teff/Tem), Ly6C^hi^CCR2^hi^CX3CR1^low^ classical monocytes, CD4+ Teff/Tem, and CD8+CD25^hi^CCR7^low^ Teff/Tem were increased by day 5. CD8+CD25^hi^CCR7^low^ Teff/Tem with the highest expression of IFN-γ, perforin, and granzyme B were enriched by day 7.

**Conclusions:**

High dimensional proteomic analysis reveals multiple innate and Teff/Tem subsets in acute rejection following VCA. In particular, IL-17A-producing γδ T cells and classical monocytes may be particularly important in initiating the alloimmune response in VCA recipients.

## 1 Introduction

Vascularized composite allotransplantation (VCA) is the transplantation of multiple tissues, including skin, muscle, bone, nerves, and blood vessels, as a single functional unit from a donor to a recipient. VCA is typically indicated for patients with severe injuries or congenital defects where traditional reconstructive surgery cannot provide satisfactory functional or aesthetic outcomes (1, 2).

According to the International Registry on Hand and Composite Tissue Transplantation, 85% of hand transplant recipients developed acute rejection within the first-year post-transplant despite rigorous immunosuppressive therapies (3). Additionally, it has been reported that 90% of hand transplant patients in the United States face similar rejection challenges (4). These reports clearly highlight that allograft rejection in VCA remains an unsolved issue. Despite its significant clinical potential, non-uterus VCA procedures remain relatively rare in the United States (5), partly due to the high incidence of rejection.

The complex nature of VCA allografts introduces unique immunological challenges to characterizing the immune responses involved in acute rejection. Therefore, elucidating the mechanisms underlying graft rejection and identifying effective therapeutic interventions are important to improve outcomes in VCA recipients. In clinical practice, patients undergoing face and hand transplants typically receive anti-thymocyte globulin induction therapy, followed by maintenance immunosuppression primarily consisting of tacrolimus, prednisone, and mycophenolate mofetil (6, 7); rejection however occurs in the majority of patients because specific underlying effector pathways of graft rejection in VCA are inadequately understood.

To address this knowledge gap, we utilized a murine hind limb allotransplant model (8, 9) to construct a dynamic atlas mapping the immune cell populations involved in acute rejection. In this study, we deepen our understanding of the immune cell subsets in VCA rejection by utilizing high dimensional, multiplexed single-cell technologies. By creating a comprehensive immune atlas, we aim to elucidate the pathways involved in VCA rejection, ultimately paving the way for improved therapeutic interventions and enhanced patient outcomes.

## 2 Materials and Methods

### 2.1 Animals

C57BL/6 (H-2K^b^) and BALB/c (H-2K^d^) male mice (8–12 weeks of age, 18–24 g) were purchased from the Jackson Laboratory (Bar Harbor, ME). Mice were housed in a temperature- and humidity-controlled environment with a 12-hour light-dark cycle under specific pathogen-free conditions and had free access to water and standard chow pellets. All experiments were conducted in accordance with the Stanford University Administrative Panel on Laboratory Animal Care which oversaw and approved all protocols (APLAC-31667).

### 2.2 Heterotopic vascularized composite allotransplantation

**Figure 1:**
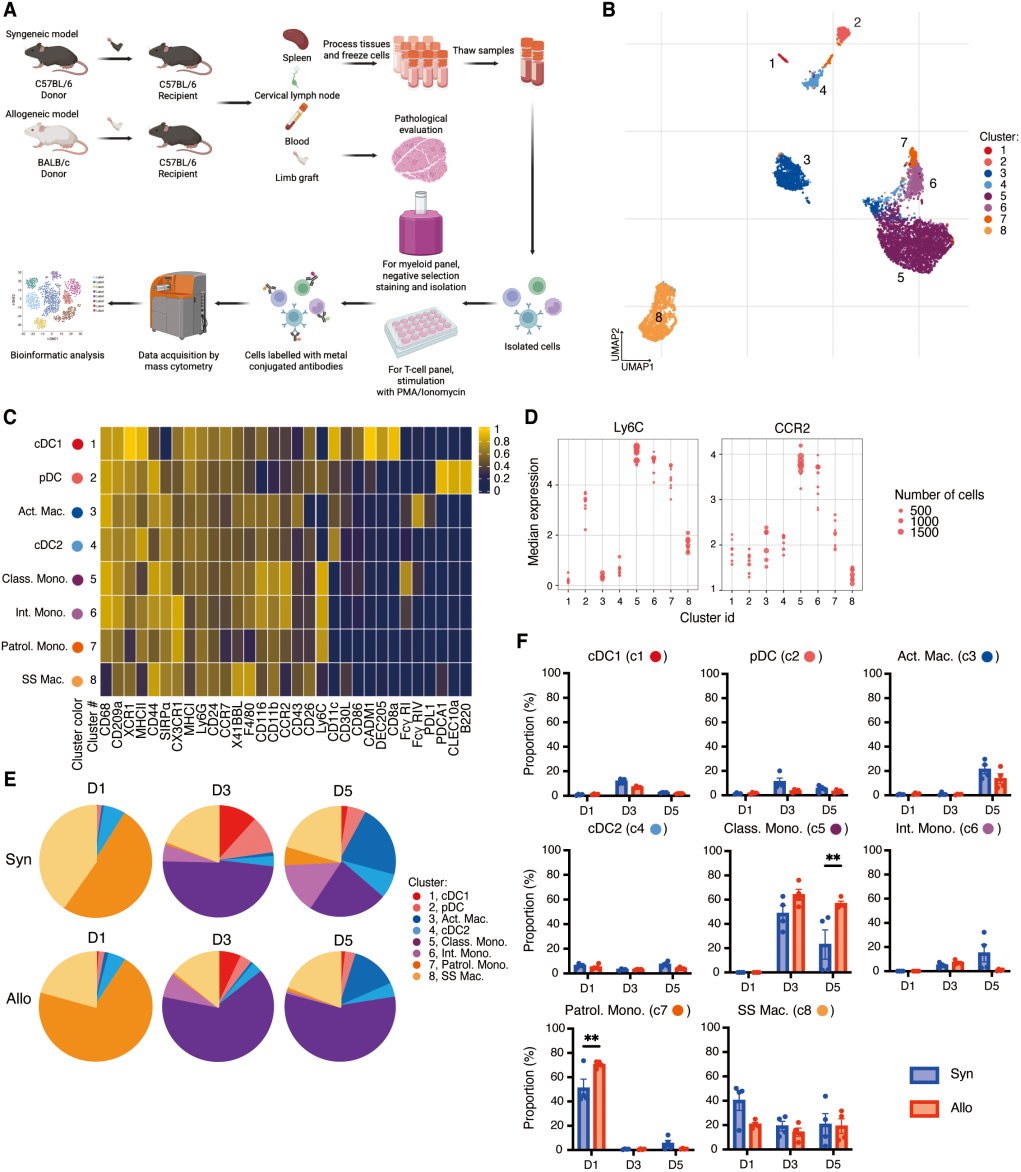
Experimental design and high dimensional analysis on myeloid cells in the spleen. **(A)**. Experimental design. This figure was created with BioRender.com. **(B)**. UMAP visualization of eight myeloid cell clusters. **(C)**. Heatmap showing that eight clusters were classified into cDC1, cDC2, pDC, classical monocytes, intermediated monocytes, patrolling monocytes, activated macrophages, and steady state macrophages according to the combination of representative surface markers. **(D)**. Clusters 5, 6, and 7 are identified as classical monocytes, intermediated monocytes, and patrolling monocytes, respectively based on the variable expression of Ly6C, CCR2. **(E)**. Pie charts showing the proportions of each cluster at three time points post-transplant. **(F)**. Proportions of each cluster at three time points after transplant. The allogeneic group showed significant increases in patrolling monocytes on day 1 and classical monocytes on day 5 compared to the syngeneic group. **: *P* < 0.01 by Sidak’s multiple comparisons test.

The details of our VCA model have been described previously (9). In brief, the donor hind limb was transplanted into the donor ipsilateral cervical area of C57BL/6 mice by anastomosing the donor femoral artery to the recipient common carotid artery and the donor femoral vein to the recipient external jugular vein. C57BL/6 mice were transplant recipients, and C57BL/6 and BALB/c mice were used as donors for syngeneic and allogeneic combinations, respectively. Samples of spleen and cervical draining lymph nodes were collected post-transplant days 1, 3, 5, and 7 (*n* =4–6 mice/group/day, **Figure 1A**). Rejection is characterized by a high degree of scarring and color change. As we have previously published (8), H&E sections from these transplanted animals demonstrated evidence of rejection by day 3, and tissue scarring and color change were present by day 5. Donor limb showed sparse inflammation in both allogeneic and syngeneic grafts on day 3, however allogeneic day 3 skin showed acute inflammation in contrast with syngeneic day 3 skin. The syngeneic day 5 limbs showed moderate degeneration and formation of dermal granulation tissue, which is associated with wound healing; however, allogeneic day 5 limbs were marked by acute inflammation and necrosis associated with rejection.

### 2.3 Tissue processing

Immediately after harvest, spleen and lymph nodes were placed in Hank’s Balanced Salt Solution (HBSS) with 2% fetal bovine serum (FBS) on ice and gently dissociated into single-cell suspensions on a Corning^®^ 70 µm cell strainer (Corning Inc., Corning, NY). After two Washes in HBSS with 2% FBS, cells were counted and cryopreserved at a concentration of 5 x 10^6^ cells/ml in 10% dimethyl sulfoxide (DMSO) with FBS using a Mr. Frosty™ Freezing Container (Thermo Fisher Scientific, Waltham, MA) in a -80°C freezer and subsequently stored in liquid nitrogen (**Figure 1A**).

### 2.4 Sample preparation and staining of myeloid cells

Splenocytes were thawed, suspended in HBSS solution containing 2% FBS, washed twice, counted, and stained with 5µM Cell-ID™ Cisplatin (Fluidigm, South San Francisco, CA) as a live/dead cell indicator. Cells were fixed using Maxpar^®^ Fix I Buffer (Fluidigm) and stained with TruStain FcX™ PLUS (anti-mouse CD16/32) antibodies (BioLegend, San Diego, CA). To isolate myeloid cells, negative selection was performed by magnetic separation using a EasySep™ Biotin Positive Selection Kit I (STEMCELL Technologies, Vancouver, Canada), targeting erythrocytes, lymphocytes, and other non-myeloid cells a with Lineage Cocktail (CD3, TCRβ, CD19, NKp46, TER119, and CD61 antibodies). Samples were barcoded with the Cell-ID™ 20-Plex Pd Barcoding Kit (Fluidigm), and all samples were combined into a single tube. The final antibody staining was conducted with a panel of metal-conjugated antibodies specific for cell surface proteins associated with myeloid cells and various activation or co-stimulatory targets (**Table 1**). After careful washing and recounting of the cells, the cells were stained with a DNA intercalator and incubated with 1.6% paraformaldehyde and Cell-ID™ Intercalator-Ir (Fluidigm) at 4° C overnight.

**Table 1:** Myeloid panel antibodies for staining and CyTOF analysis.

Epitope	Tag	Clone	Source	Cat. No.
CD45	89 Y	30-F11	Fluidigm	3089005B
Ly6C	111 Cd	HK1.4	Biolegend	128002
SIRPa (CD172a)	112 Cd	P84	Biolegend	144002
LINEAGE	113 In			
CD3e		145-2C11	Biolegend	100303
TCRβ		H57-597	Biolegend	109203
CD19		6D5	Biolegend	115503
NKp46		29A1.4	Biolegend	137615
TER119 (Erythroid cells)		TER-119	Biolegend	116203
CD61		2C9.G2	Biolegend	104304
CD26	114 Cd	H194-112	Biolegend	137802
PDCA-1 (CD317)	115 In	927	Biolegend	127002
CX3CR1	116 Cd	SA011F11	Biolegend	149002
CD24	140 Ce	M1/69	Biolegend	101802
Ly6G	141 Pr	1A8	Fluidigm	3141008B
CD11c	142 Nd	N418	Fluidigm	3142003B
DEC-205 (CD205)	143 Nd	NLDC-145	Biolegend	138202
MHC Class I	144 Nd	28-14-8	Fluidigm	3144016B
Fcγ RII/III	144 Nd	93	Biolegend	101302
MERTK	145 Nd	2B10C42	Biolegend	151502
CD43	146 Nd	S11	Fluidigm	3146009B
CD45.2	147 Sm	104	Fluidigm	3147004B
CD11b	148 Nd	M1/70	Fluidigm	3148003B
CD103	149 Sm	M290	BD Pharmigen	553699
CLEC9a	150 Nd	7H11	Biolegend	143502
Fcγ RI	151 Eu	X54-5/7.1	Fluidigm	3151012B
OX40L	152 Gd	RM134L	Biolegend	108802
CD8a	153 Eu	53-6.7	Fluidigm	3153012B
CD45.1	153 Eu	A20	Fluidigm	3153002B
MGL2 (CD301b)	154 Gd	URA-1	Biolegend	146802
CD163	155 Gd	S150491	Biolegend	155302
CD70	155 Gd	FR70	BD Biosciences	562226
ICOSL	156 Gd	HK5.3	Biolegend	107405
Fcγ RIV	157 Gd	9E9	Biolegend	149502
CCR2	158 Gd	475301R	R & D Systems	MAB55381R
F4.80	159 Tb	BM8	Fluidigm	3159009B
CD116	160 Gd	698423	Thermo Fisher Scientific	MA5-23918
4-1BBL (TNFSF9)	161 Dy	203942	R & D Systems	MAB1246
CD40	161 Dy	HM40-3	Fluidigm	3161020B
CD30L (CD153)	162 Dy	RM153	Biolegend	106409
XCR1	163 Dy	ZET	Biolegend	148202
CCR7 (CD197)	164 Dy	4B12	Fluidigm	3164013A
CD44	165 Ho	IM7	Biolegend	103002
CD68	166 Er	FA-11	Biolegend	137002
CD326 (EpCAM)	166 Er	G8.8	Fluidigm	3166014B
CD209a	167 Er	MMD3	Biolegend	833001
H-2Dd	167 Er	34-2-12	Biolegend	110602
CD21	168 Er	7G6	Fisher Scientific	BDB562796
PDL1 (CD274)	169 Tm	MIH7	Biolegend	155402
CD169 (Siglec-1)	170 Er	3D6.112	Fluidigm	3170018B
CD80	171 Yb	16-10A1	Fluidigm	3171008B
CD86	172 Yb	GL1	Fluidigm	3172016B
CLEC10a	173 Yb	LOM-8.7	Biolegend	145602
YAe	174 Yb	eBioY-Ae	Thermo Fisher Scientific	11-5741-82
CADM1	175 Lu	3E1	MBL International	CM004-3
B220 (CD45R)	176 Yb	RA3-6B2	Fluidigm	3176002B
MHC Class II (I-A/I-E)	209 Bi	M5/114.15.2	Fluidigm	3209006B

### 2.5 Sample preparation and staining of T cells

Cells from spleen and lymph nodes were thawed and suspended in 200 µg/µL of DNase (Worthington Biochemical Corporation, Lakewood, NJ)-added complete RPMI, consisting of RPMI 1640 (Cytiva, Marlborough, MA) with 10% FBS, 1% penicillin-streptomycin (Gibco, Waltham, MA), 1% GlutaMX supplement (Gibco), and 1% HEPES (Gibco). After washing, the cells were counted, rested in complete RPMI without DNase for 30 minutes at 37° C in a 5% CO_2_ incubator. The samples were stimulated with 1x Cell Stimulation Cocktail (eBioscience, San Diego, CA), containing phorbol 12-myristate 13-acetate (PMA) and ionomycin, and 1x Protein Transport Inhibitor Cocktail (eBioscience), containing Brefeldin A and Monensin for five hours at 37° C in a 5% CO_2_ incubator. After the stimulation, cells were stained with 5µM Cell-ID™ Cisplatin for live/dead cell discrimination After fixation and permeabilization with eBioscience™ Foxp3/Transcription Factor Staining Buffer Set, the samples were barcoded with the Cell-ID™ 20-Plex Pd Barcoding Kit, and all samples were combined into a single tube. The combined sample was stained with TruStain FcX™ PLUS (anti-mouse CD16/32) antibody, followed by staining of membrane proteins with heavy metal-conjugated antibodies. After fixation and permeabilization, cells were stained for intracellular proteins with heavy metal-conjugated antibodies and incubated with 1.6% paraformaldehyde and Cell-ID™ Intercalator-Ir (Fluidigm) at 4°C overnight.

### 2.6 CyTOF and data analysis

Samples were analyzed on a Helios™ mass cytometer. After two washes, the samples were suspended in a 10% EQ Four Element Calibration Beads (Fluidigm) with Cell Acquisition Solution (Fluidigm). After running the samples, Flow Cytometry Standard (FCS) files were normalized, concatenated, and debarcoded using the Helios software. CellEngine (http://cellengine.com) was used to identify target lymphocytes as follows (8, 10). Briefly, cellular events were identified using a DNA-intercalator dye (191-iridium and 193-iridium double positive). Singlets were extracted from all cellular events based on event length, and live cells were extracted from singlets based on cisplatin staining (195-cisplatin negative). Live cells were extracted from CellEngine for the myeloid panel and live CD3+ cells for the T cell panel as new.fsc files and imported into RStudio (v4.3.3) for further analysis. We utilized an R-based CyTOF analysis workflow (11). Markers used for clustering were chosen based on prior knowledge combined with a non-redundancy score that ranks markers by their contribution to between-sample variability (11). For analysis of myeloid cells, we first classified the cells into 12 clusters and removed non-myeloid cells, such as T cells, B cells, NK cells, granulocytes, and then re-classified the remaining cells into 8 populations. For analysis of T cells, the cells were classified into 18 populations. Median marker expressions of clusters were visualized with heatmaps, and uniform manifold approximation and projection (UMAP) was used for dimensionality reduction and visualization of clusters.

### 2.7 Statistical analysis

Differences among experimental and control groups were analyzed using two-way analysis of variance (ANOVA), followed by Sidak’s multiple comparisons test to assess inter-group differences at each time point. *P*<0.05 was considered statistically significant (*: *P*<0.05, **: *P*<0.01, ***: *P*<0.001, ****: *P*<0.0001). All statistical analyses were performed with Prism 10 (Graph Pad Software, Inc., San Diego, CA) and R 4.3.3 (https://cran.r-project.org/).

## 3 Results

### 3.1 Patrolling monocytes and Ly6C^hi^CCR2^hi^CX3CR1^low^ classical monocytes are increased early post-transplant in the spleen of allogeneic VCA recipients

We previously demonstrated that VCA is characterized by the early emergence of an Ly6C^hi^/CD62L^+^ inflammatory monocyte population and CD8^+^granzyme B^+^ T cell subset in the periphery of allograft recipients (8), suggesting rapid and vigorous innate and adaptive immune cell collaboration. To create a comprehensive and dynamic immune atlas of acute rejection in VCA (BALB/c limb onto C57BL/6 recipient), we performed deep immune phenotyping of myeloid and T cell populations in spleen and lymph nodes on days 1, 3, and 5 post-transplant using custom antibody panels and mass cytometry (**Figure 1A**).

Splenocytes were collected and stained with a panel of 45 antibodies to resolve myeloid cell subsets. Using uniform manifold approximation and projection (UMAP), we identified eight cell clusters that encompass the major myeloid populations (**Figure 1B**). As shown in the heatmap (**Figure 1C**), these eight clusters were classified according to the combination of representative surface markers. Cluster 1, distinguished by CD11c^+^XCR1^hi^CD11b^-^MHCII^+^, represents conventional dendritic cells 1 (cDC1), while Cluster 4 expressing CD11c^+^XCR1^low^CD11b^+^MHCII^+^ is classified as conventional dendritic cells 2 (cDC2). Cluster 2 is distinguished by low expression of CD11c, high expression of PDCA1 and B220, and represents plasmacytoid dendritic cell (pDC). Clusters 3 and 8 are consistent with macrophages, demonstrating high expression of CD11b and F4/80 and the absence of Ly6C. Cluster 3 are activated macrophages based on expression of FcγR IV^hi^ and PD-L1^+^, while Cluster 8, which lacks FcγR IV and PD-L1, are steady state macrophages. Clusters 5, 6, and 7 are monocytes with variable Ly6C expression (**Figure 1D**) and are further classified as classical monocytes, intermediate monocytes, and patrolling monocytes, respectively based on the expression of Ly6C, CCR2, and CX3CR1 (**Figure 1C**) (12–14). Among the three clusters of monocytes, cluster 5 showed the highest expression of Ly6C and CCR2, while cluster 7 had the lowest expression of Ly6C and CCR2, and cluster 6 had intermediate levels of CCR2 and Ly6C (**Figure 1D**). In addition, cluster 5 expressed the lowest level of CX3XR1 (**Figure 1C**). The proportions of each cluster in the spleen at three time points post-transplant are summarized in **Figures 1E, F**. On day 1 post-transplant, patrolling monocytes (cluster 7) and steady state macrophage (cluster 8) were the predominant myeloid populations in the spleen. Patrolling monocytes (cluster 7) were significantly (*P*=0.002) increased in the allogeneic group as compared to the syngeneic group on day 1. There were small, transient increases of cDC1 (cluster 1) on day 3 and small increases of pDC (cluster 2) on day 3 and 5 post-transplant but these changes did not significantly differ between the syngeneic and allogeneic groups. Similarly, there were increases in activated macrophages on day 5 (cluster 3) and sustained increases of steady state macrophages beginning on day 1 (cluster 8) but there were no significant differences between the syngeneic and allogeneic groups (**Figure 1F**). Classical monocytes (cluster 5), which were scarce on day 1 post-transplant, increased on day 3 and were significantly (*P*=0.003) elevated in the spleens of the allogeneic group as compared to the syngeneic group on day 5. Give that classical monocytes can give rise to intermediate monocytes, the late increase of intermediate monocytes (cluster 6) on day 5 in the syngeneic group suggests they may have transitioned from the syngeneic classical monocytes that were transiently increased on day 3.

Overall, these data indicate that patrolling monocytes are the predominant myeloid population in the spleen early post-transplant and are replaced by classical monocytes with significant increases in allogeneic recipients.

### 3.2 CD8+CD25^hi^CCR7^low^ effector/effector memory T cells are increased in the spleen of VCA recipients

To achieve a parallel, high dimensional analysis of the T cell response associated with rejection of VCA allografts, splenocytes from groups of syngeneic and allogeneic recipients (**Figure 1A**) were stimulated with PMA/ionomycin for five hours, stained with a panel of antibodies and then analyzed by mass cytometry to assess the expression of 18 surface and 9 intracellular proteins (**Table 2**). Murine T cells are most commonly categorized on the basis of the expression of CD44, CD62L, and CCR7, and described as naïve T cell (Tnaïve, CD44^low^CCR7^hi^), Teff/Tem (CD44^hi^CD62L^low^), central memory T cell (CD44^hi^CD62L^hi^), and regulatory CD4+ T cell (Treg, CD4+Foxp3^hi^).

**Table 2:** T cell panel antibodies for staining and CyTOF analysis.

Epitope	Tag	Clone	Source	Cat. No.	ECS or ICS
CD4	116 Cd	RM4-5	Fluidigm	92J004116	ECS
CD44	141 Pr	IM7	Fluidigm	92J005141	ECS
GITR	143 Nd	DTA-1	Fluidigm	3143019B	ECS
IL-2	144 Nd	JES6-5H4	Fluidigm	3144002B	ICS
CD69	145 Nd	H1.2F3	Fluidigm	3145005B	ECS
IL-17A	148 Nd	TC11-18H10.1	Biolegend	506935	ICS
CD25	150 Nd	3C7	Fluidigm	3150002B	ECS
CD28	151Eu	37.51	Biolegend	102119	ECS
CD3e	152 Sm	145-2C11	Fluidigm	3152004B	ECS
CD8a	153 Eu	53-6.7	Fluidigm	3153012B	ECS
CTLA-4 (CD152)	154 Sm	UC10-4B9	Fluidigm	3154008B	ECS
CD27	155 Gd	A18209B	Biolegend	110102	ECS
CCR6 (CD196)	156 Gd	29-2L17	Fluidigm	3156016A	ECS
Foxp3	158 Gd	FJK-16s	Fluidigm	3158003A	ICS
H-2Dd	159 Tb	34-2-12	Biolegend	110602	ECS
CD62L	160 Gd	MEL-14	Fluidigm	3160008B	ECS
TNF-α	162 Dy	MP6-XT22	Fluidigm	3162002B	ICS
CCR7 (CD197)	164 Dy	4B12	Fluidigm	3164013A	ECS
IFN-γ	165 Ho	XMG1.2	Fluidigm	3165003B	ICS
IL-10	166 Er	JES5-16E3	Biolegend	505029	ICS
IL-6	167 Er	MP5-20F3	Fluidigm	3167003B	ICS
TCRβ	169 Tm	H57-597	Fluidigm	3169002B	ECS
CD40L (CD154)	170 Er	MR1	Fluidigm	3170011B	ECS
TCRγδ	172 Yb	GL3	Fluidigm	92J014172	ECS
Granzyme B	173 Yb	GB11	Fluidigm	3173006B	ICS
LAG3 (CD223)	174 Yb	C9B7W	Fluidigm	3174019B	ECS
Perforin	175 Lu	S16009A	Biolegend	154302	ICS

ECS, extracellular staining; ICS intracellular staining.

**Figure 2:**
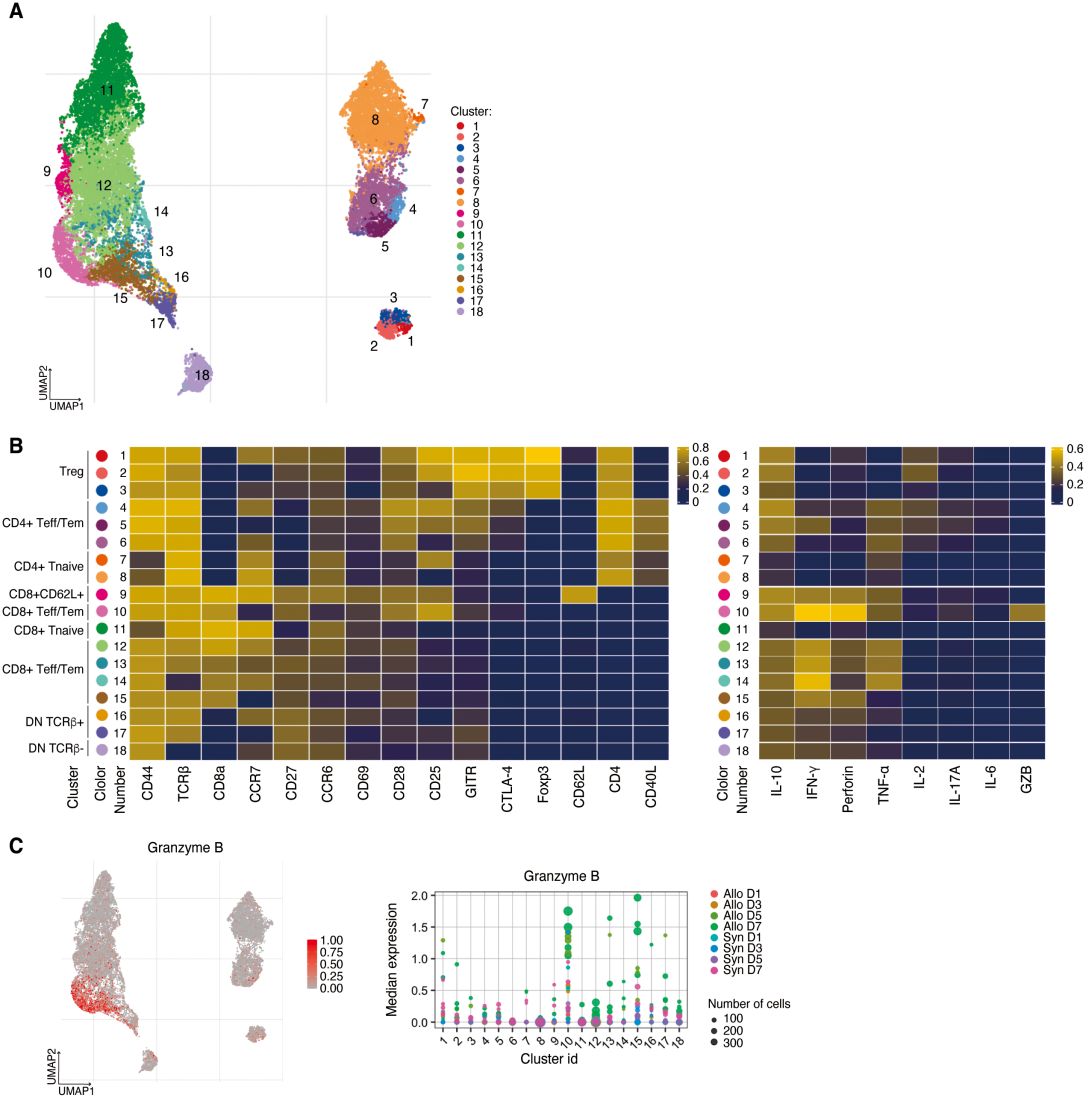
High dimensional analysis on T cells in the spleen. **(A)** UMAP visualization of T cells classified into 18 clusters. **(B)** Left, heatmap showing the expressions of surface markers in Treg, CD4+/CD8+ Tnaïve, CD4+ and CD8+ Teff/Tem, CD8+CD62L+ T cells, and DN T cells. Right, heatmap demonstrating their expressions of cytokines, perforin, and granzyme B **(C)** Left, UMAP visualization of Granzyme B expression. Right, CD8+CD25^hi^CCR7^low^ Teff/Tem (cluster 10) are distinguished from other effector T cell populations by increased Granzyme B expression.

After gating on live CD3+ cells, a UMAP of 18 clusters was generated (**Figure 2A**), including Treg, CD4+/CD8+ Tnaïve, CD4+ and CD8+ Teff/Tem, CD8+CD62L+ T cells, and double negative (DN) T cells (**Figure 2B**). CD4+ T cells are represented in clusters 1–8, CD8+ T cells in clusters 9–15, and DN T cells in clusters 16–18. Clusters 1–3 represent Foxp3+ Tregs with differential expression levels of CD25 and CCR7. Clusters 4–6 are CD4+ CD44^hi^CD62L^low^Teff/Tem distinguished primarily by CD25 and CCR7 expression, with cluster 5 having higher IFN-γ expression than the other CD4+ Teff/Tem, while cluster 6 has lower IL-2 expression (**Figure 2B**). CD8+CD62L+ T cells were identified in cluster 9. CD8+ Teff/Tem were classified into five clusters (clusters 10, 12–15), marked by higher expression of IFN-γ than found in the other clusters. IFN-γ strongly promotes innate and inflammatory responses and is associated with graft rejection (15–17). In particular, CD8+CD25^hi^CCR7^low^ Teff/Tem (cluster 10) exhibited the highest expression of IFN-γ, and perforin compared to the other CD8+ Teff/Tem and was further distinguished from other effector T cell populations by increased granzyme B expression suggesting it has potent cytotoxic function (**Figures 2B, C**).

**Figure 3:**
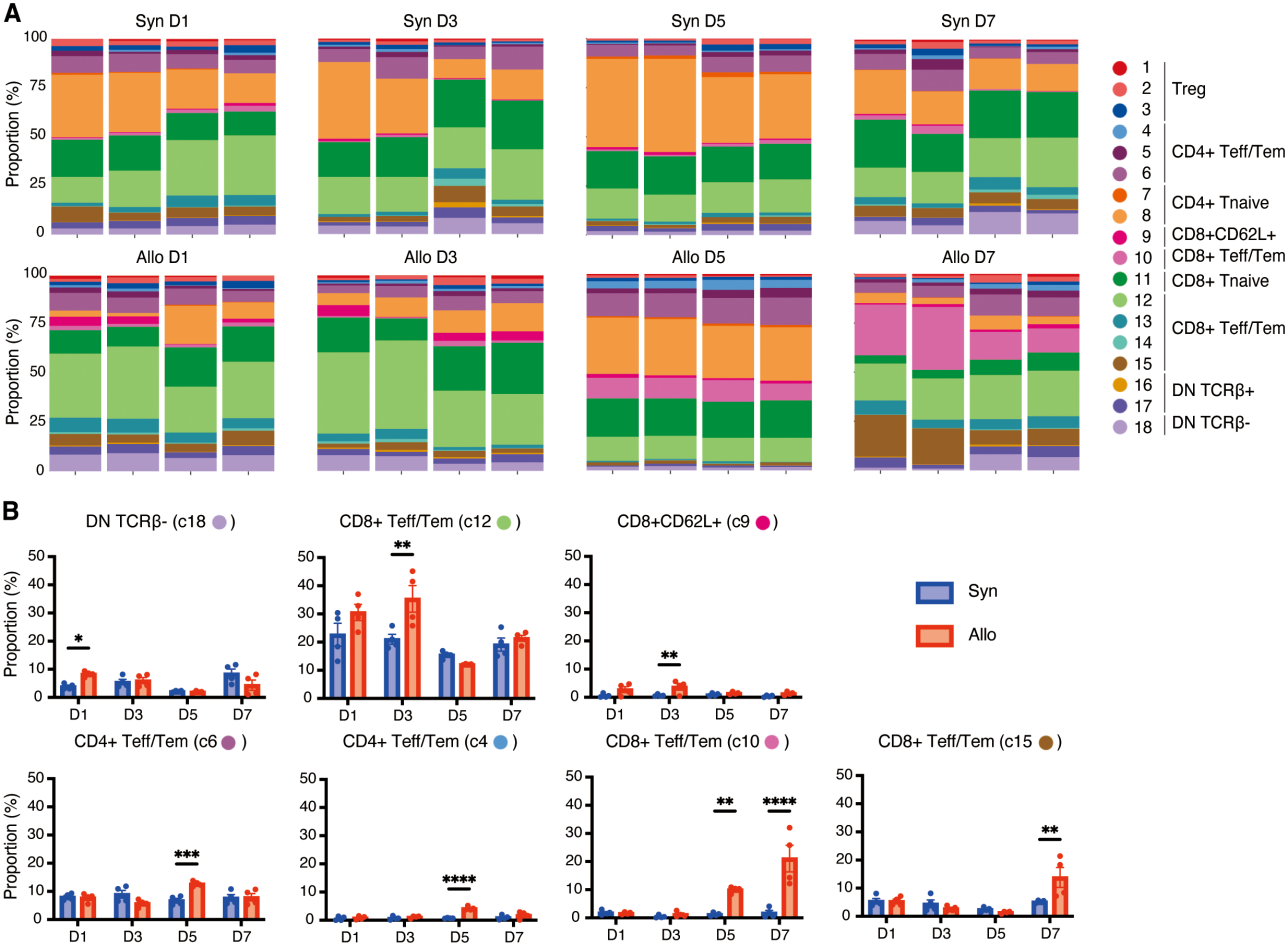
Dynamic changes of T-cell clusters in the spleen following VCA. **(A)**. Proportions of each cluster in each sample at four time points following transplant. **(B)**. Bar graphs showing the proportions of the seven clusters at four time points post-transplant. Allogeneic recipients exhibited the early expansion of DN TCRβ- T cells (cluster 18) followed by CD8+ Teff/Tem (cluster 12) and CD8+CD62L+ T cells (cluster 9). Three distinct CD4+/CD8+ Teff/Tem clusters (clusters 6, 4, and 10) were increased on day 5 in allograft recipients, and the increase of CD8+CD25^hi^CCR7^low^ Teff/Tem (cluster 10) in allograft recipients persisted through day 7 and was accompanied by marked expansion of CD8+CD25^low^CCR7^low^ Teff/Tem (cluster 15). *: *P* < 0.05, **: *P* < 0.01, ***: *P* < 0.001, ****: *P* < 0.0001 by Sidak’s multiple comparisons test.

To assess the dynamics of T cell populations during the post-transplant period we analyzed longitudinal changes in the proportion of T cell clusters in the spleen (**Figures 3A, B**). On day 1 post-transplant, DN TCRβ- T cells (cluster 18) were significantly increased in the allogeneic group (*P*<0.001). By day 3, the predominant CD8+ T cell populations were CD8+ Teff/Tem (cluster 12) and CD8+CD62L+ T cells (cluster 9), with these populations significantly more abundant in the allogeneic group compared to the syngeneic group (*P*=0.003 and *P*=0.005, respectively).

In contrast, we noted subsequent increases in three distinct CD4+/CD8+ Teff/Tem clusters beginning on day 5 (clusters 6, 4, and 10) that were significantly increased in allograft recipients (*P*<0.001, *P*<0.001, *P*=0.006, respectively). Increases in CD8+CD25^hi^CCR7^low^ Teff/Tem (cluster 10) in allograft recipients persisted through day 7 (*P*<0.001) and were accompanied by marked expansion of CD8+CD25^low^CCR7^low^ Teff/Tem (cluster 15) in the allogeneic group (*P*=0.001). T cells in cluster 15 showed lower expression of CD25, IFN-γ, perforin, and granzyme B compared to CD8+CD25^hi^CCR7^low^ Teff/Tem (cluster 10) (**Figure 2B**), suggesting that CD8+CD25^hi^CCR7^low^ Teff/Tem (cluster 10) transitioned to CD8+CD25^low^CCR7^low^ Teff/Tem (cluster 15).

Together these data indicate that VCA is characterized by a very early expansion of DN T cells in the spleen followed by a rapid increase in two populations of CD8+ T cells, one Teff/Tem population expressing IFN-γ, perforin and another, CD8+CD62L+ population expressing IFN-γ, TNF-α, and perforin. The most potent effector cell emerges in allograft recipients on day 5, peaks on day 7, and is CD8+CD27+CD28+CD25+, expressing high IFN-γ, perforin, and granzyme B. The CD4+ T cell response is slightly delayed by comparison and is marked by two CD4+CD28+CCR7+CD40L+TNF-α+ Teff/Tem populations that peak on day 5 and differ by CD25, CTLA-4, and IL-2 expression. Thus, the T cell response in VCA is rapid, dynamic, and dominated by CD8+ T cells.

### 3.3 IL-17 expressing double negative T cells emerge in allogeneic recipient lymph nodes very early after transplant

**Figure 4:**
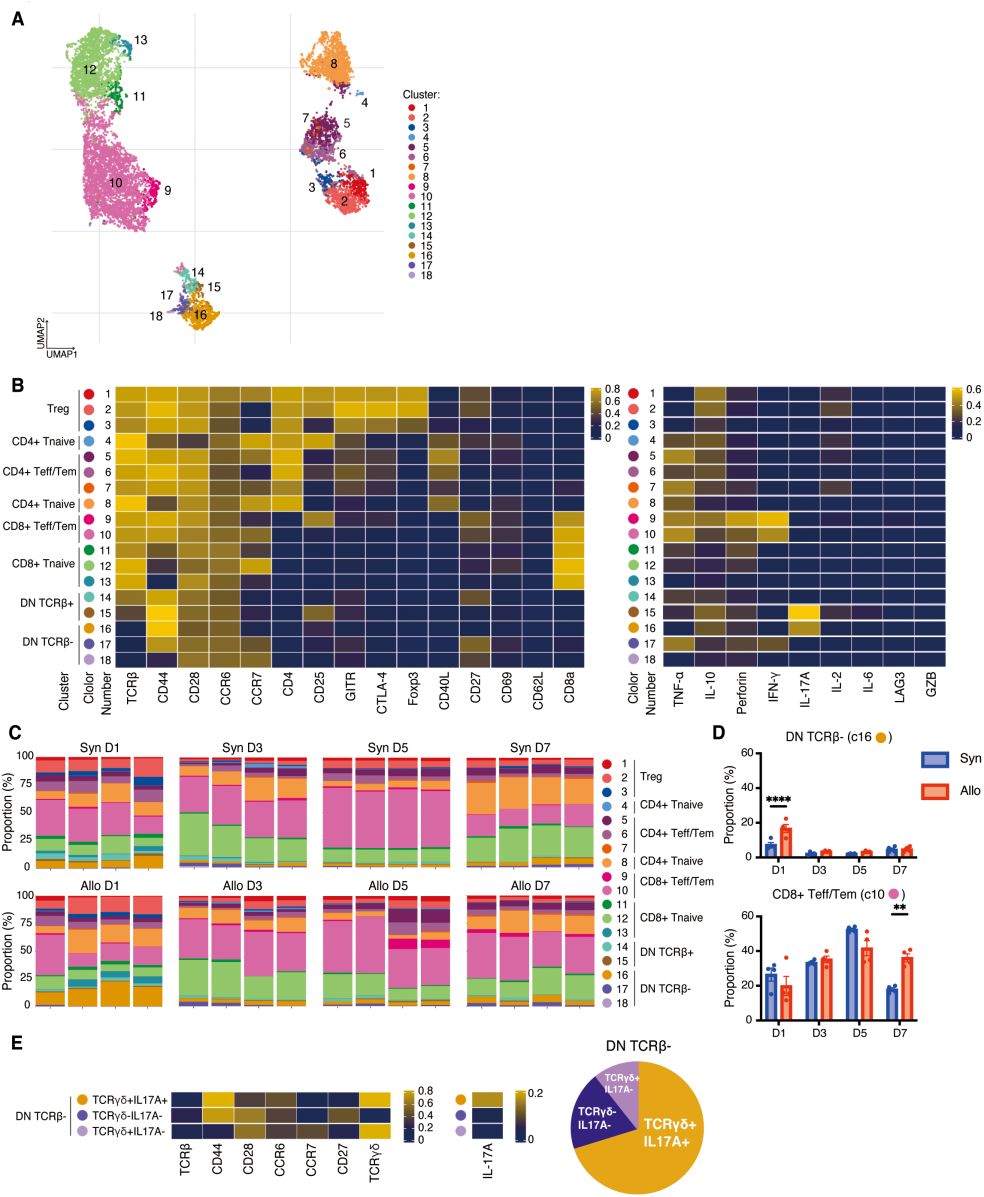
High dimensional analysis on T cells in the draining lymph nodes. **(A)**. UMAP visualization of T cells classified into 18 clusters. **(B)**. Left, heatmap showing the expressions of surface markers in Treg, CD4+/CD8+ Tnaïve, CD4+ and CD8+ Teff/Tem, CD8+CD62L+ T cells, and DN T cells. Right, heatmap demonstrating their expressions of cytokines, perforin, and granzyme B, **(C)**. Proportions of each cluster in each sample at four time points post-transplant. The predominant population in the lymph nodes were CD8+CD25^low^CCR7^low^ Tem (cluster 10). **(D)**. The allogeneic group showed significant increases in DN TCRβ- T cells (cluster 16) on day 1 and CD8+CD25^low^CCR7^low^ Tem (cluster 10) on day 7 compared to the syngeneic group. **: *P* < 0.01, ****: *P* < 0.0001 by Sidak’s multiple comparisons test. **(E)**. Additional experiments presented that cluster 16 corresponded to TCRγδ+IL17A+, cluster 17 to TCRγδ-IL17A-, and cluster 18 to TCRγδ+IL17A- cells.

To detect potential differences between systemic and local immune response, and to compare the response to VCA in different lymphoid tissues, single-cell suspensions were prepared from the draining lymph nodes on days 1, 3, 5, and 7 post-transplant and stimulated with PMA/ionomycin as described above. After gating on live CD3+ cells, a UMAP was generated for 18 populations (**Figure 4A**). As shown in **Figure 4B**, eight clusters of CD4+ T cells, consisting of naive cells (clusters 4, 8), effector/memory (clusters 5–7), and regulatory T cells (clusters 1–3) are indicated on the heatmap while five clusters of CD8+ T cells, including naive cells (clusters 11–13) and effector/memory cells (clusters 9, 10) were identified. The predominant population in the lymph nodes were CD8+CD25^low^CCR7^low^ Tem (cluster 10) which showed high expression of IFN-γ and perforin and was significantly increased in recipients of allografts on day 7 post-transplant (*P*=0.001, **Figures 4C, D**).

In addition, five distinct clusters of DN T cells were identified (clusters 14–18), amongst which clusters 16–18 were characterized by TCRβ- populations (**Figure 4B**). Cluster 16 (CD44^hi^CCR6^hi^CCR7^low^CD27-) represented the largest proportion of the DN TCRβ- T cells (**Figures 4A, C**). To further investigate DN TCRβ-T cells, we analyzed the draining lymph nodes on day 1 post-transplant by incorporating an anti-TCRγδ antibody in our CyTOF panel. By comparing the expression of IL-17A, CD44, CD27, CCR6, and CCR7 in **Figure 4B** with their respective expression in **Figure 4E**, we determined that cluster 16 corresponded to TCRγδ+IL17A+, cluster 17 to TCRγδ-IL17A-, and cluster 18 to TCRγδ+IL17A- cells (**Figure 4E**). Cluster 16 (DN TCRβ-IL-17A+ T cells) showed a significant increase on day 1 in draining lymph nodes of the allogeneic group compared to the syngeneic group (*P*<0.001, **Figure 4D**). This finding suggests that IL-17A-expressing γδ T cells may initiate a rapid alloimmune response in recipients of VCA.

## 4 Discussion

**Figure 5:**
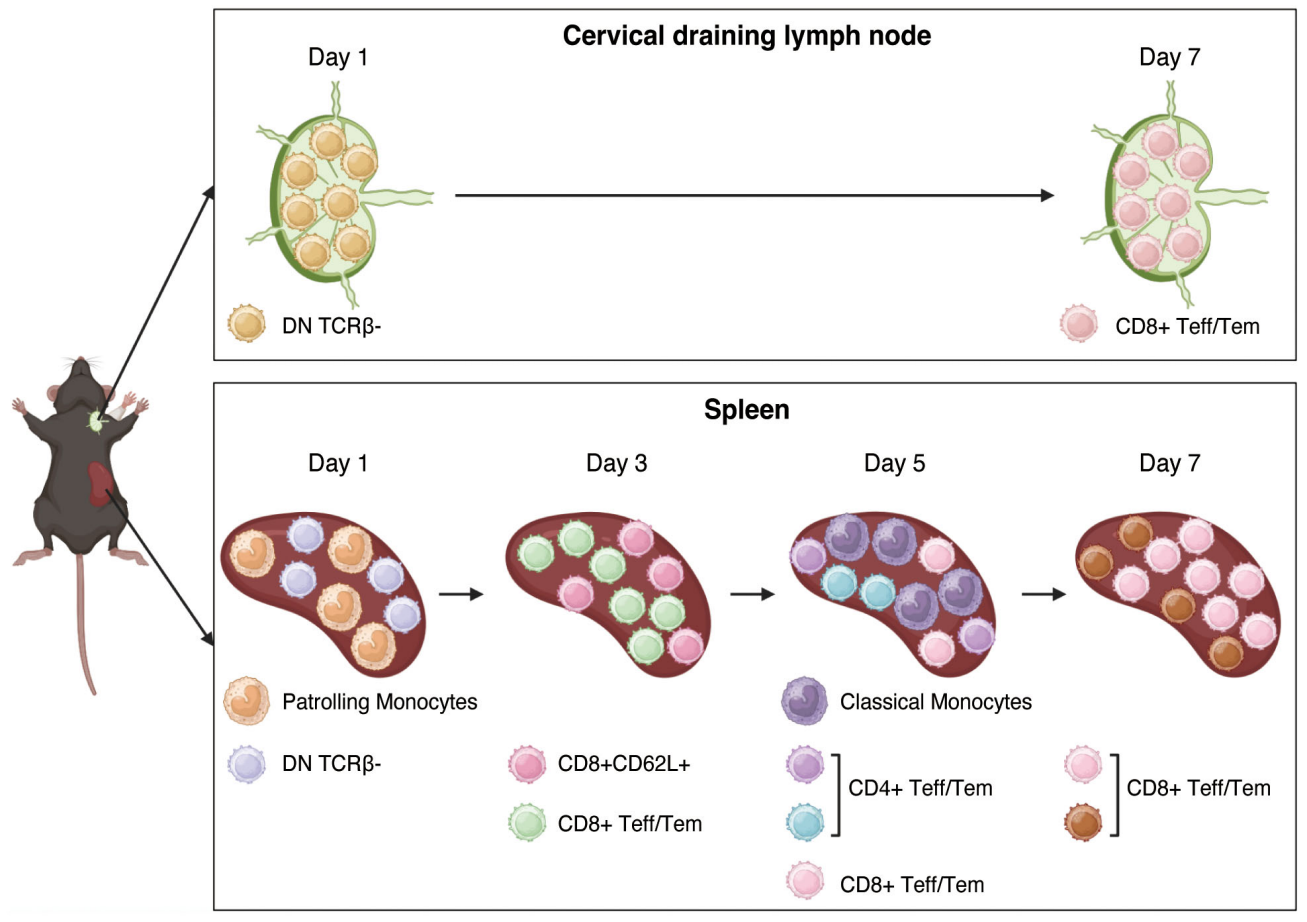
Summary of the cell populations associated with rejection in VCA allografts. In the very early post-transplant period, DN TCRβ- T cells, including IL-17A+ γδ T cells, were significantly elevated in the spleen and draining lymph nodes of allogeneic recipients, and patrolling monocytes were abundant in the allogeneic spleen. Subsequently, CD8+CD62L+ T cells and CD8+ Teff/Tem cells were significantly increased in the spleens of allogeneic recipients compared to syngeneic recipients, followed by significant increases in classical monocytes, CD4+ Teff/Tem cells, and CD8+ Teff/Tem cells in the allogeneic spleen. Thereafter, CD8+ Teff/Tem cells expressing the highest levels of IFN-γ, perforin, and granzyme B became significantly more prevalent in the allogeneic spleen than in the syngeneic spleen. This figure was created with BioRender.com.

The clinical success of VCA has been hampered by the high incidence of acute rejection despite the use of intensive immunosuppressive regimens. Thus, it is important to develop more specific immunomodulatory approaches based on an improved mechanistic understanding of the immune response to composite tissue grafts. To elucidate the specific cell types responsible for initiating and mediating acute graft rejection in a murine hind limb model of VCA, we applied high-dimensional CyTOF analysis. This comprehensive approach enabled us to identify immune cell populations associated with graft rejection and create a temporal map of the immune response following VCA (**Figure 5**). Very early after transplant, DN TCRβ- T cells were significantly increased in both the spleen and draining lymph nodes of allogeneic recipients, and patrolling monocytes were prevalent in the spleen of allogeneic recipients. Subsequently, CD8+CD62L+ T cells and CD8+ Teff/Tem were significantly increased in the spleen of allogeneic recipients compared to syngeneic recipients, followed by significant increases in classical monocytes, CD4+ Teff/Tem, and CD8+ Teff/Tem in the spleens of allogeneic recipients. Thereafter, CD8+ Teff/Tem with the highest expression of IFN-γ, perforin, and granzyme B were significantly increased.

To date, experimental research in animal models of VCA has mainly focused on evaluating the efficacy of immunosuppressive therapies. Studies utilizing anti-CD4, anti-CD8, and anti-CD40L antibodies as key components of immunosuppressive strategies have demonstrated graft survival of 67 days (18) and over 200 days (19) post-transplantation in mice. Additionally, a combination of anti-CD45RB antibody and rapamycin has been shown to extend graft survival to 100 days (20). These findings indicate that robust systemic immunosuppressive regimens can lead to prolonged graft survival in mouse models. In contrast, several studies have explored the use of both systemic and localized immunosuppressive therapies. One study reported graft survival exceeding 300 days using microparticles containing TGF-β1, IL-2, and rapamycin as a drug delivery system, combined with systemic immunosuppression with tacrolimus and antilymphocyte serum (21). Fisher et al. achieved more than 200 days of graft survival with a regimen of tacrolimus, antilymphocyte serum, and microparticles containing CCL22 (22). These findings underscore the potential efficacy of localized immunosuppressive strategies, which are particularly applicable to VCA. We found an early increase in DN TCRβ- T cells, followed by an increase in CD8+ Teff/Tem in the draining lymph nodes, and in the spleen, suggesting that the regulation of local immune responses may be important in VCA. In addition, given that immunosuppressive strategies both in experimental models (18–20) and clinical practice (6, 7, 23) have focused on controlling conventional T cells, our findings are important because they showed that IL-17A-producing γδ T cells and classical monocytes increase very early after transplantation and are not specifically targeted by current immunosuppressive regimens. While there may be interactions between γδ T cells and classical monocytes, it is also possible that they are involved independently. Further investigation will be necessary to clarify their roles and elucidate the mechanisms of rejection in VCA.

Of interest, DN TCRβ- T cells exhibited a specific increase in allogeneic recipients very early after transplantation. Our results suggest that IL-17A+ DN T cells present in the lymph node are a subset of γδ T cells. Various γδ T cell subsets, including Vγ 1, 4, 5, 6, and 7, have been reported (24). Among these, the murine Vγ4 subset, characterized by IL-17A+CD44+CCR6+ expression, is known to localize to lymph nodes (24). The phenotype of the DN T cells were identified in the lymph nodes of VCA recipients (IL-17A+CD44^hi^CCR6^hi^CD27-), align with those of the Vγ4 subset. IL-17A+ γδ T cells are recognized for their role in the early stages of the inflammatory response (25). Our studies show that γδ T cells may be a sentinel T cell in VCA. Indeed, IL-17A is a key mediator of tissue inflammation in various autoimmune diseases and transplantation. A direct role for γδ T cells in T-cell mediated rejection has been demonstrated in a murine model of heart transplantation. This role is primarily associated with their production of IL-17A, which accelerates rejection by inhibiting Treg expansion (26). Furthermore, studies using a murine model of skin transplant showed that IL-17A-producing γδ T cells may contribute to the accumulation of mature DCs in draining lymph nodes (27), thereby regulating the function of αβ T cells and facilitating the cross-priming of CD8+ T cells (27, 28). These mechanisms may also be relevant to the pathogenesis of rejection in VCA.

Focusing on myeloid cells, we identified monocyte dynamics associated with VCA rejection. Monocytes represent a heterogeneous population of immune cells involved in a range of inflammatory processes (29–31). Patrolling monocytes are present in both homeostatic and inflamed tissues and perform a specialized form of immune homeostasis. They initiate acute inflammatory responses and contribute to tissue remodeling, partly through their ability to engage in extensive intravascular crawling or patrolling, which allows them to rapidly respond to inflammatory stimuli (32, 33). Classical monocytes, on the other hand, are typically recruited from the circulation to inflammatory sites, in many cases *via* their high expression of CCR2, where they produce inflammatory cytokines and differentiate into proinflammatory macrophages (34). This study demonstrated a significant increase in patrolling monocytes very early post-transplant, followed by an expansion of classical monocytes in allogeneic recipients, suggesting that patrolling monocytes were involved in the early response to alloantigen, with the majority subsequently being replaced by classical monocytes. Furthermore, in unpublished studies we have demonstrated that the absence of CCR2 contributes to significantly improved allograft survival. Consequently, Ly6C^hi^CCR2^hi^CX3CR1^low^ classical monocytes were significantly associated with allograft rejection in VCA.

While DN TCRβ- T cells were the earliest T cells shown to expand in allograft recipients, there were subsequent increases in CD8+CD62L+ T cells and CD8+ Teff/Tem in the spleens of allogeneic recipients. CD8+CD62L+ T cells are likely to differentiate into central memory T cells due to their high expression of CD62L (35). CD8+ Teff/Tem which increased along with CD8+CD62L+ T cells expressed relatively low levels of cytokines, whereas CD8+CD25^hi^CCR7^low^ Teff/Tem, which were significantly increased in allogeneic recipients later, showed the highest expression of IFN-γ, perforin, and granzyme B. Additionally, CD4+ Teff/Tem showed a significant increase in allogeneic recipients at the timing when CD8+CD25^hi^CCR7^low^ Teff/Tem began to increase. These findings suggest that CD8+ Teff/Tem with low cytokine expression increased early after transplantation, while more cytotoxic CD8+ Teff/Tem expanded later, likely with the assistance of CD4+ Teff/Tem.

While CyTOF is a highly powerful tool for high-dimensional single-cell analysis, it does have certain limitations (36, 37). For example, issues related to antibody specificity and sensitivity may arise, potentially affecting the accuracy of marker detection. In addition, data drop-outs—where low-abundance proteins may not be consistently detected—and clustering biases introduced during computational analysis can influence the interpretation of cellular subsets and differentiation trajectories. Moreover, CyTOF is based on single-cell suspensions and therefore inherently lacks spatial context. For understanding critical spatial relationships between immune cells and tissue architecture, such as graft infiltration and immune cell localization, complementary technologies like spatial transcriptomics and imaging mass cytometry can be highly valuable (38–40). The combined use of CyTOF with these spatial approaches has the potential to yield more effective and comprehensive analytical results.

In conclusion, the comprehensive approach using CyTOF enabled us to identify unexpected immune cell populations associated with rejection in VCA. Therapeutics that specifically target IL-17A-producing γδ T cells and classical monocytes may be particularly effective in controlling rejection in VCA recipients.

## Data Availability

The raw data supporting the conclusions of this article will be made available by the authors, without undue reservation.

## References

[1] Jones JW Gruber SA Barker JH Breidenbach WC. Successful hand transplantation. One-year follow-up. Louisville Hand Transplant Team. N Engl J Med. (2000) 343:468–73. doi: 10.1056/NEJM200008173430704 10950668

[2] Dubernard JM Lengele B Morelon E Testelin S Badet L Moure C. Outcomes 18 months after the first human partial face transplantation. N Engl J Med. (2007) 357:2451–60. doi: 10.1056/NEJMoa072828 18077810

[3] Petruzzo P Lanzetta M Dubernard JM Landin L Cavadas P Margreiter R. The international registry on hand and composite tissue transplantation. Transplantation. (2010) 90:1590–4. doi: 10.1097/TP.0b013e3181ff1472 21052038

[4] Hein RE Ruch DS Klifto CS Leversedge FJ Mithani SK Pidgeon TS. Hand transplantation in the United States: A review of the Organ Procurement and Transplantation Network/United Network for Organ Sharing Database. Am J Transplant. (2020) 20:1417–23. doi: 10.1111/ajt.15704 31733027

[5] Hernandez JA Miller JM Emovon E 3rd Howell JN Testa G Israni AK. OPTN/SRTR 2022 annual data report: vascularized composite allograft. Am J Transplant. (2024) 24:S534–S56. doi: 10.1016/j.ajt.2024.01.020 38431366

[6] Homsy P Huelsboemer L Barret JP Blondeel P Borsuk DE Bula D. An update on the survival of the first 50 face transplants worldwide. JAMA Surg. (2024) 159(12):1339–45. doi: 10.1001/jamasurg.2024.3748 PMC11411447 39292472

[7] Wells MW Rampazzo A Papay F Gharb BB. Two decades of hand transplantation: A systematic review of outcomes. Ann Plast Surg. (2022) 88:335–44. doi: 10.1097/SAP.0000000000003056 35113506

[8] Harden JT Wang X Toh J Sang AX Brown RA Esquivel CO. High-resolution phenotyping of early acute rejection reveals a conserved alloimmune signature. Cell Rep. (2021) 34:108806. doi: 10.1016/j.celrep.2021.108806 33657374 PMC12662566

[9] Wang X Harden JT Sang AX Esquivel CO Martinez O Krams SM. Establishment of heterotopic hind limb transplantation model in the mouse. Transplant Proc. (2021) 53:491–4. doi: 10.1016/j.transproceed.2020.10.039 33341263

[10] Rao M Amouzgar M Harden JT Lapasaran MG Trickey A Armstrong B. High-dimensional profiling of pediatric immune responses to solid organ transplantation. Cell Rep Med. (2023) 4:101147. doi: 10.1016/j.xcrm.2023.101147 37552988 PMC10439249

[11] Nowicka M Krieg C Crowell HL Weber LM Hartmann FJ Guglietta S. CyTOF workflow: differential discovery in high-throughput high-dimensional cytometry datasets. F1000Res. (2017) 6:748. doi: 10.12688/f1000research.11622.3 28663787 PMC5473464

[12] Yang J Zhang L Yu C Yang XF Wang H. Monocyte and macrophage differentiation: circulation inflammatory monocyte as biomarker for inflammatory diseases. Biomark Res. (2014) 2:1. doi: 10.1186/2050-7771-2-1 24398220 PMC3892095

[13] Mildner A Marinkovic G Jung S. Murine monocytes: origins, subsets, fates, and functions. Microbiol Spectr. (2016) 4. doi: 10.1128/microbiolspec.MCHD-0033-2016 27780020

[14] Montgomery AB Chen SY Wang Y Gadhvi G Mayr MG Cuda CM. Tissue-resident, extravascular Ly6c(-) monocytes are critical for inflammation in the synovium. Cell Rep. (2023) 42:112513. doi: 10.1016/j.celrep.2023.112513 37204925 PMC10697497

[15] Ivashkiv LB. IFNgamma: signalling, epigenetics and roles in immunity, metabolism, disease and cancer immunotherapy. Nat Rev Immunol. (2018) 18:545–58. doi: 10.1038/s41577-018-0029-z PMC6340644 29921905

[16] Obara H Nagasaki K Hsieh CL Ogura Y Esquivel CO Martinez OM. IFN-gamma, produced by NK cells that infiltrate liver allografts early after transplantation, links the innate and adaptive immune responses. Am J Transplant. (2005) 5:2094–103. doi: 10.1111/j.1600-6143.2005.00995.x PMC1473982 16095488

[17] Lin CM Plenter RJ Coulombe M Gill RG. Interferon gamma and contact-dependent cytotoxicity are each rate limiting for natural killer cell-mediated antibody-dependent chronic rejection. Am J Transplant. (2016) 16:3121–30. doi: 10.1111/ajt.13865 PMC5083186 27163757

[18] Tung TH Mackinnon SE Mohanakumar T. Prolonged limb allograft survival with CD 40 costimulation blockade, T-cell depletion, and megadose donor bone-marrow transfusion. Microsurgery. (2005) 25:624–31. doi: 10.1002/micr.20170 16281278

[19] Li Z Benghiat FS Charbonnier LM Kubjak C Rivas MN Cobbold SP. CD8+ T-Cell depletion and rapamycin synergize with combined coreceptor/stimulation blockade to induce robust limb allograft tolerance in mice. Am J Transplant. (2008) 8:2527–36. doi: 10.1111/j.1600-6143.2008.02419.x 18853957

[20] Zhong T Liu Y Jiang J Wang H Temple CL Sun H. Long-term limb allograft survival using a short course of anti-CD45RB monoclonal antibody, LF 15-0195, and rapamycin in a mouse model. Transplantation. (2007) 84:1636–43. doi: 10.1097/01.tp.0000290277.23186.ad 18165776

[21] Fisher JD Balmert SC Zhang W Schweizer R Schnider JT Komatsu C. Treg-inducing microparticles promote donor-specific tolerance in experimental vascularized composite allotransplantation. Proc Natl Acad Sci U S A. (2019) 116:25784–9. doi: 10.1073/pnas.1910701116 PMC6925993 31792185

[22] Fisher JD Zhang W Balmert SC Aral AM Acharya AP Kulahci Y. *In situ* recruitment of regulatory T cells promotes donor-specific tolerance in vascularized composite allotransplantation. Sci Adv. (2020) 6:eaax8429. doi: 10.1126/sciadv.aax8429 32201714 PMC7069700

[23] Testa G McKenna GJ Wall A Bayer J Gregg AR Warren AM. Uterus transplant in women with absolute uterine-factor infertility. JAMA. (2024) 332(10):817–24. doi: 10.1001/jama.2024.11679 PMC11327905 39145955

[24] Ribot JC Lopes N Silva-Santos B. gammadelta T cells in tissue physiology and surveillance. Nat Rev Immunol. (2021) 21:221–32. doi: 10.1038/s41577-020-00452-4 33057185

[25] Papotto PH Ribot JC Silva-Santos B. IL-17(+) gammadelta T cells as kick-starters of inflammation. Nat Immunol. (2017) 18:604–11. doi: 10.1038/ni.3726 28518154

[26] Li Y Huang Z Yan R Liu M Bai Y Liang G. Vgamma4 gammadelta T cells provide an early source of IL-17A and accelerate skin graft rejection. J Invest Dermatol. (2017) 137:2513–22. doi: 10.1016/j.jid.2017.03.043 28733202

[27] Rahimpour A Mattarollo SR Yong M Leggatt GR Steptoe RJ Frazer IH. gammadelta T cells augment rejection of skin grafts by enhancing cross-priming of CD8 T cells to skin-derived antigen. J Invest Dermatol. (2012) 132:1656–64. doi: 10.1038/jid.2012.16 PMC3352982 22358058

[28] Kaminski H Couzi L Eberl M. Unconventional T cells and kidney disease. Nat Rev Nephrol. (2021) 17:795–813. doi: 10.1038/s41581-021-00466-8 34446934

[29] Geissmann F Jung S Littman DR. Blood monocytes consist of two principal subsets with distinct migratory properties. Immunity. (2003) 19:71–82. doi: 10.1016/s1074-7613(03)00174-2 12871640

[30] Sunderkotter C Nikolic T Dillon MJ Van Rooijen N Stehling M Drevets DA. Subpopulations of mouse blood monocytes differ in maturation stage and inflammatory response. J Immunol. (2004) 172:4410–7. doi: 10.4049/jimmunol.172.7.4410 15034056

[31] Tan Z Hall P Mack M Snelgrove SL Kitching AR Hickey MJ. Both classical and non-classical monocytes patrol glomerular capillaries and promote acute glomerular inflammation. Am J Pathol. (2024) 195(1):89–101. doi: 10.1016/j.ajpath.2024.07.010 39117108

[32] Hanna RN Cekic C Sag D Tacke R Thomas GD Nowyhed H. Patrolling monocytes control tumor metastasis to the lung. Science. (2015) 350:985–90. doi: 10.1126/science.aac9407 PMC4869713 26494174

[33] Finsterbusch M Hall P Li A Devi S Westhorpe CL Kitching AR. Patrolling monocytes promote intravascular neutrophil activation and glomerular injury in the acutely inflamed glomerulus. Proc Natl Acad Sci U S A. (2016) 113:E5172–81. doi: 10.1073/pnas.1606253113 PMC5024581 27528685

[34] Woollard KJ Geissmann F. Monocytes in atherosclerosis: subsets and functions. Nat Rev Cardiol. (2010) 7:77–86. doi: 10.1038/nrcardio.2009.228 20065951 PMC2813241

[35] Sallusto F Geginat J Lanzavecchia A. Central memory and effector memory T cell subsets: function, generation, and maintenance. Annu Rev Immunol. (2004) 22:745–63. doi: 10.1146/annurev.immunol.22.012703.104702 15032595

[36] Krams SM Schaffert S Lau AH Martinez OM. Applying mass cytometry to the analysis of lymphoid populations in transplantation. Am J Transplant. (2017) 17:1992–9. doi: 10.1111/ajt.14145 PMC5526773 27888565

[37] Zhang W Sen A Pena JK Reitsma A Alexander OC Tajima T. Application of mass cytometry platforms to solid organ transplantation. Transplantation. (2024) 108:2034–44. doi: 10.1097/TP.0000000000004925 PMC11390974 38467594

[38] Angelo M Bendall SC Finck R Hale MB Hitzman C Borowsky AD. Multiplexed ion beam imaging of human breast tumors. Nat Med. (2014) 20:436–42. doi: 10.1038/nm.3488 PMC4110905 24584119

[39] Keren L Bosse M Marquez D Angoshtari R Jain S Varma S. A structured tumor-immune microenvironment in triple negative breast cancer revealed by multiplexed ion beam imaging. Cell. (2018) 174:1373–87 e19. doi: 10.1016/j.cell.2018.08.039 30193111 PMC6132072

[40] Veenstra J Dimitrion P Yao Y Zhou L Ozog D Mi QS. Research techniques made simple: use of imaging mass cytometry for dermatological research and clinical applications. J Invest Dermatol. (2021) 141:705–12 e1. doi: 10.1016/j.jid.2020.12.008 33752807 PMC7995633

